# Mutational Analysis of the Yeast TRAPP Subunit Trs20p Identifies Roles in Endocytic Recycling and Sporulation

**DOI:** 10.1371/journal.pone.0041408

**Published:** 2012-09-26

**Authors:** Hichem Mahfouz, Antonella Ragnini-Wilson, Rossella Venditti, Maria Antonietta De Matteis, Cathal Wilson

**Affiliations:** 1 Telethon Institute of Genetics and Medicine, Naples, Italy; 2 Department of Biology, University of Rome “Tor Vergata”, Rome, Italy; 3 Consorzio Mario Negri Sud, Santa Maria Imbaro, Chieti, Italy; Simon Fraser University, Canada

## Abstract

Trs20p is a subunit of the evolutionarily conserved TRAPP (TRAnsport Protein Particle) complex that mediates various aspects of membrane trafficking. Three TRAPP complexes have been identified in yeast with roles in ER-to-Golgi trafficking, post-Golgi and endosomal-to-Golgi transport and in autophagy. The role of Trs20p, which is essential for viability and a component of all three complexes, and how it might function within each TRAPP complex, has not been clarified to date. To begin to address the role of Trs20p we generated different mutants by random mutagenesis but, surprisingly, no defects were observed in diverse anterograde transport pathways or general secretion in Trs20 temperature-sensitive mutants. Instead, mutation of Trs20 led to defects in endocytic recycling and a block in sporulation/meiosis. The phenotypes of different mutants appear to be separable suggesting that the mutations affect the function of Trs20 in different TRAPP complexes.

## Introduction

The transport of proteins and lipids via small vesicles or larger tubulovesicular structures from the endoplasmic reticulum (ER) to the Golgi and thence to intracellular compartments or the plasma membrane (PM) is a complex and highly regulated process. The efficient transport of cargo proteins requires that compartmental identity be maintained by controlling the spatio-temporal flux of membranes and proteins between compartments by both anterograde and retrograde trafficking processes. SNARE proteins (soluble N-ethylmaleimide-sensitive factor attachment protein receptors) present in the donor and acceptor membranes mediate membrane fusion and show compartment-specific localization that confers specificity on the fusion reaction [1]. However, other factors, called tethering factors, are important in conferring specificity in membrane-membrane pairing at a stage before the SNARE-mediated fusion reaction. Two broad classes of molecules are proposed to have a role in tethering, a group of coiled-coil proteins (such as GM130 and p115) and several large, multi-subunit complexes (for example, the TRAPP and COG complexes) [2]. One of the best-characterized tethering factors is the TRAPP complex. Yeast cells have three TRAPP complexes: TRAPP I contains seven subunits (Bet3 (two copies), Bet5, Trs31, Trs33, Trs23, and Trs20), TRAPP II contains all of the TRAPP I subunits plus an additional three (Trs65, Trs120 and Trs130), while TRAPP III contains TRAPP I plus one additional subunit, Trs85 [3], [4], [5]. Recently, additional TRAPP subunits have been identified: a Trs20-like protein (Tca17/TRAPPC2L) present in both yeast and mammals that appears to be part of TRAPP II [6], [7] and mammalian-specific subunits C4orf41 and TTC-15 that operate in ER-to-Golgi trafficking [8].

TRAPP I is required for the fusion of vesicles with the Golgi by tethering COPII vesicles via a direct interaction between Bet3p and Sec23p, a component of the COP II coat [9]. TRAPP I is also a guanine nucleotide-exchange factor (GEF) for the small GTPase Ypt1p (only the Bet3, Bet5, Trs23 and Trs31 subunits are required for this activity) [10], [11], while TRAPP II may act as a GEF for the small GTPases Ypt31p/32p [12]. The TRAPP II-specific components Trs130p and Trs120p have been implicated in post-Golgi transport and early endosome-to-Golgi trafficking, respectively [13]. Consistent with TRAPP II having a function at the late Golgi are the genetic and functional interactions of Trs130, Trs33, and Trs65 with the small GTPases Ypt31/32 and the phosphatidylinositol-4-kinase Pik1 [14], [15], which regulate exocytic/endocytic trafficking at the trans-Golgi. TRAPP III, on the other hand, has been shown to have a role in autophagy [4]. The TRAPP subunits are conserved from yeast to humans but by contrast with the different TRAPP complexes that can be identified in yeast only a single high-molecular weight complex has been identified in mammals to date [8].

Mutations of the TRAPP component sedlin, the mammalian orthologue of Trs20p, cause spondyloepiphyseal dysplasia tarda (SEDT), a late-onset skeletal genetic disorder [16]. Expression of the sedlin gene in yeast cells deleted for Trs20, an essential gene, can suppress the lethality illustrating conservation of function [17]. Trs20p has been reported to be a component of all three TRAPP complexes where it occupies a position on the periphery of each complex, but is not required for the GEF activity. Structural studies of the TRAPP II complex suggested a role for Trs20p in linking the TRAPP I complex to a TRAPP II-specific component such as Trs130p [18]. However, the role of Trs20p in trafficking or as part of the TRAPP complex is largely unknown. The crystal structure of sedlin [19] identified it as a longin-domain protein, a domain identified in several SNARE proteins (Ykt6p and Sec22p) that may mediate protein-protein interactions. Here we performed an unbiased mutational screen of Trs20 and identified a number of mutation-dependent phenotypes that may reflect its diverse roles as part of the different TRAPP complexes.

## Results

### Generation of Trs20 and Bet3 mutants

Although deletion of the Trs20 gene results in lethality, previous studies found that neither strong repression of Trs20 expression nor reducing Trs20 mRNA stability led to any overt phenotype or defects in CPY trafficking to the vacuole [17], [20]. We therefore undertook a screen to isolate temperature-sensitive (ts) mutants of Trs20 to gain insights into its physiological function.

**Figure 1:**
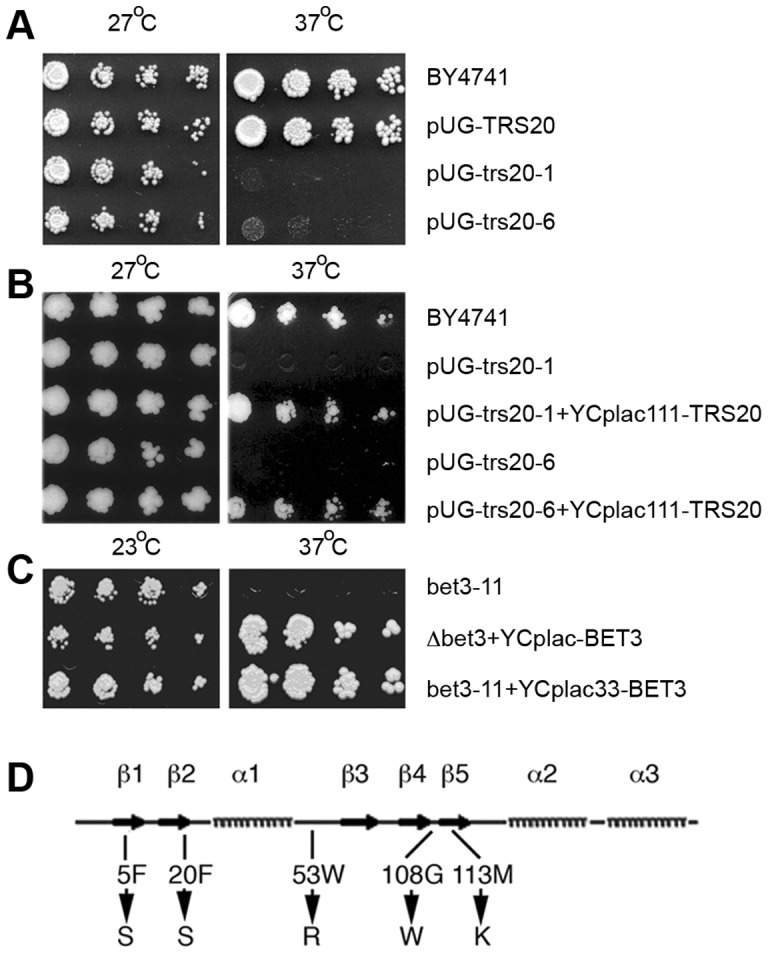
Growth phenotypes of *pUG-trs20* and *bet3-11* mutants. Serial dilutions were spotted onto YPD plates and incubated for 3 days at the indicated temperatures. (A) Temperature-sensitive phenotype of Trs20 mutants (Δtrs20 cells containing plasmid pUG23-trs20-1 or pUG23-trs20-6) compared to cells expressing a WT copy of Trs20 (Δtrs20 cells containing plasmid pUG23-Trs20) and BY4741 (isogenic cells containing the chromosomal copy of the Trs20 gene). (B) The temperature-sensitive phenotype of the Trs20 mutants is complemented by co-expression of a WT copy of Trs20 from the plasmid YCplac111. (C) The temperature-sensitive phenotype of the *bet3-11* allele (plasmid YCplac111-bet3-11 in Δbet3 cells) is complemented by co-expressing a WT copy of Bet3 from the plasmid YCplac33. (D) Location of amino acid substitutions in *pUG-trs20-1* and *pUG-trs20-6*. The secondary structure is of the mouse sedlin protein [19], but the amino acid positions refer to the yeast protein.

In an initial screen, temperature-sensitive mutants were generated using error-prone PCR combined with plasmid shuffling (see Materials and Methods). As templates for the error-prone PCR reactions we used both a Trs20 gene construct under its own promoter and terminator or a construct containing the Trs20 gene with a Met25 promoter and a GFP tag (YCplac111-TRS20 and pUG23-Trs20, respectively, see Table S2). However, temperature-sensitive mutants were isolated only from the latter screen. The two that showed the strongest ts phenotype (called *pUG-trs20-1* and *pUG-trs20-6*, Figure 1A) were selected for further study. The ts phenotypes could be complemented by co-expressing a wild-type version of the gene from plasmid YCplac111-TRS20 (Figure 1B) showing that the temperature sensitivity was indeed due to mutations in the Trs20 gene. The mutant proteins are encoded by plasmid-borne GFP-tagged constructs in a Δtrs20 background (deleted for the Trs20 gene), so Δtrs20 cells expressing the GFP-tagged wild-type (WT) Trs20 protein (herein referred to as *pUG-TRS20*), which fully complements deletion of the Trs20 gene (Figure 1A), were used as a control, unless otherwise stated. While all GFP-tagged Trs20 proteins are functional, in that they rescue the deletion of the Trs20 gene, they produce only a weak diffuse fluorescence pattern whether tagged with GFP at the amino or carboxy terminus, in the presence or absence of the mutations or when assayed at different temperatures or in different media (Figure S1 and data not shown). Western analysis suggested that the temperature sensitivity is probably due to misfolding/steric hindrance rather than degradation of the GFP-tagged mutant proteins (Figure S2).

Sequencing of the inserts revealed that each construct contained more than one mutation. The *pUG-trs20-1* mutant contained a phenylalanine to serine mutation at position 5 (F5S) in the β1 sheet and a tryptophan to arginine mutation at position 53 (W53R) after the first α helix (Figure 1D) [19]. The *pUG-trs20-6* mutant had undergone three mutations; F20S (within β2), G108W (between β4 and β5), and M113K (within β5) (Figure 1D). These residues, except for G108, are conserved between yeast and humans. The β-sheet mutations are within the structural hydrophobic core of the protein while the W53R mutation is within a putative protein interaction domain [19]. The G108 residue is substituted by a surface-exposed histidine residue in the mouse sedlin protein [19].

Mutation of Bet3, a core element of all of the TRAPP complexes, has been shown to block intracellular transport [3], [21]. We therefore generated a Bet3 ts mutant to be used as a control in trafficking assays of the Trs20 mutants. The Bet3 gene was cloned into the plasmid YCplac111 and a ts mutant was generated using site-directed mutagenesis of two previously described mutated residues in Bet3p (see Materials and Methods). The growth of this ts mutant, *bet3-11*, was retarded even at 27°C so further experiments were conducted using 23°C as the permissive temperature. This ts phenotype could be rescued by co-expressing a WT copy of the Bet3 gene cloned in the plasmid YCplac33 (Figure 1C).

### The Trs20 mutants do not show defects in diverse anterograde trafficking pathways

As Trs20 has been reported to be a subunit of the TRAPP I complex, which mediates ER-to-Golgi trafficking [21], we first investigated whether the *pUG-trs20-1* and *pUG-trs20-6* mutants affect transport between the ER and the Golgi, the localization of Golgi marker proteins or trafficking to the vacuole and the plasma membrane. We took advantage of the absence of any fluorescence pattern of the tagged Trs20 WT and mutant proteins (Figure S1) to analyze GFP-tagged reporter molecules representing different classes of molecules and various trafficking pathways. The *bet3-11* mutant, the TRAPP II mutant strain VSY446 (*trs130-HA^ts^*) [15], and other well-characterized trafficking mutants such as *sec23-1* (COPII), *sec21-3* (COPI) and *sec18-1* (NSF) (see Table S1), were used for comparative purposes.

#### ER-to-Golgi transport

**Figure 2:**
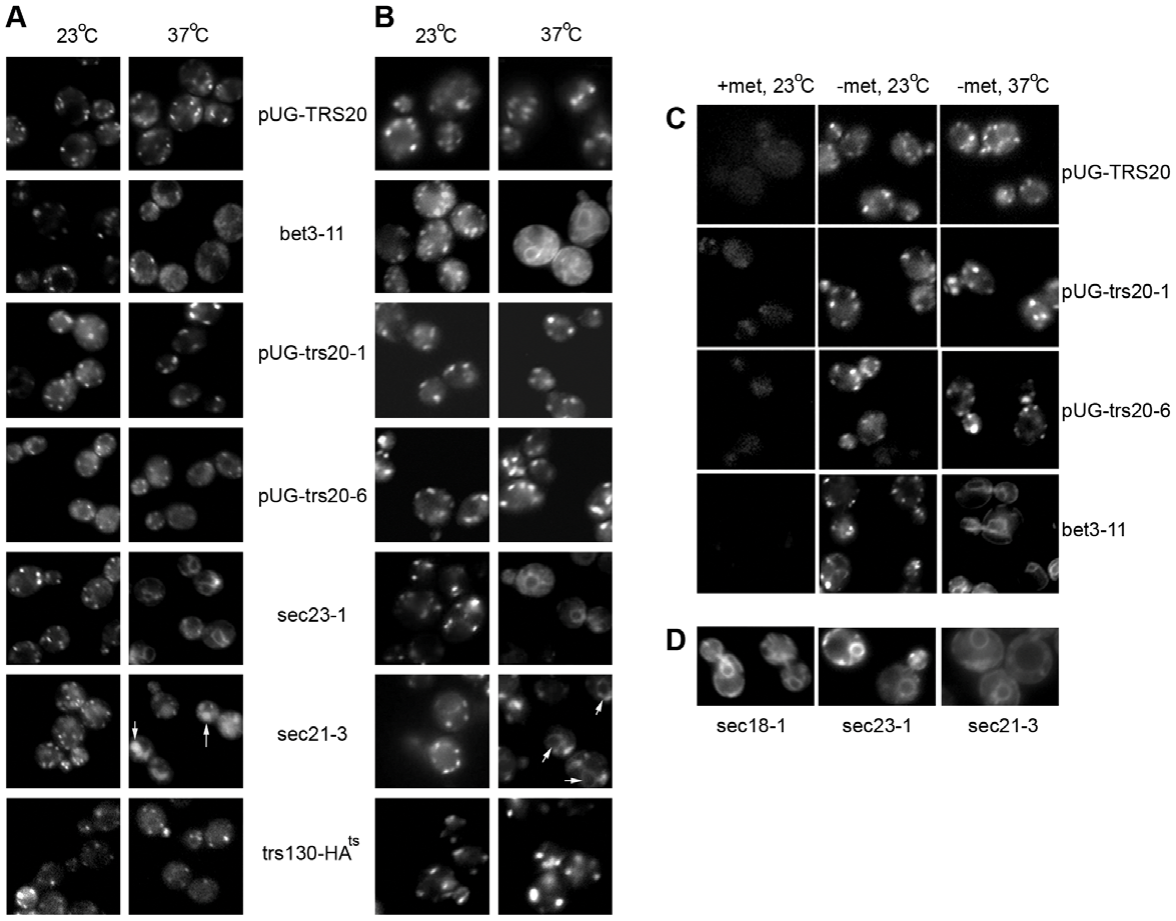
GFP-Rer1p and GFP-Gos1p localization is unaffected in *pUG-trs20-1* and *pUG-trs20-6*. The indicated yeast strains transformed with (**A**) pSKY5 (that expresses GFP-tagged Rer1p) or (**B**) pUG34-Gos1 (that expresses GFP-tagged Gos1p) were observed by fluorescence microscopy at 23°C or after being shifted to 37°C for 30 minutes. In (A) white arrows indicate missorting of GFP-Rer1p to the vacuole in *sec21-3* cells. In (B) the *sec21-3* cells show both a punctate Golgi and a perinuclear ER fluorescence pattern (white arrows) at the restrictive temperature. (**C**) The indicated strains expressing GFP-Gos1p under control of the methionine-suppressible Met25 promoter were grown in medium containing 1 mM methionine (left panels), then washed and resuspended in methionine-free medium at 23°C (middle panels) or 37°C (right panels) for 30 minutes. (**D**) The indicated strains expressing GFP-Gos1p were grown in methionine-containing medium, washed and resuspended in methionine-free medium at 37°C for 30 minutes.

Rer1p is a receptor protein that cycles between the ER and the Golgi but under steady-state conditions localizes principally on the cis-Golgi exhibiting a typical punctate Golgi-like pattern. Blocking ER exit in the COPII mutant *sec23-1* resulted in the accumulation of a GFP-Rer1 fusion protein in the ER while in the COPI mutant *sec21-3* it was missorted to the vacuole (Figure 2A), in accordance with previous reports [22], [23]. In the *bet3-11* mutant, the punctate pattern of GFP-Rer1p was dispersed (Figure 2A) as might be expected due to the disassembly of the Golgi in Bet3 mutants [21]. However, the GFP-Rer1p fluorescence pattern was not markedly affected in either of the Trs20 mutants or in the *trs130-HA^ts^* mutant (Figure 2A), suggesting that the Trs20 mutants do not affect cis-Golgi structure or the recycling pathway of Rer1p from the Golgi to the ER.

We next tested whether the localization pattern of a medial-Golgi marker protein, the SNARE protein Gos1p [24], is affected using a GFP-tagged Gos1 protein. In the COPII mutant *sec23-1* the punctate GFP-Gos1p pattern became diffuse at the restrictive temperature (Figure 2B) while it was unaffected in the *sec21-3* COPI mutant (Figure 2B), as expected [24], [25]. The *bet3-11* mutant showed a fluorescence pattern similar to the *sec23-1* mutant (Figure 2B) while the punctate pattern of GFP-Gos1p was not markedly affected in the *pUG-trs20-1, pUG-trs20-6* or *trs130-HA^ts^* mutants (Figure 2B), similarly to *pUG-TRS20* cells.

We noted the presence of a perinuclear fluorescence pattern in the *sec23-1, sec21-3* and *bet3-11* mutants at the restrictive temperature (Figure 2B) suggesting the possibility that newly synthesised GFP-Gos1p might be blocked in exiting the ER. To test this we took advantage of the fact that GFP-Gos1p expression is driven by the methionine-suppressible Met25 promoter on the plasmid pUG36. No GFP-Gos1p fluorescence was observed when *pUG-TRS20* cells were grown in medium containing 1 mM methionine, but rapid synthesis occurred after resuspending the cells in methionine-free medium resulting in the typical punctate Golgi pattern both at 23°C and 37°C (Figure 2C). Using this induction protocol, GFP-Gos1p was readily observed as a punctate Golgi pattern in the *bet3-11* mutant at 23°C but a perinuclear ER pattern resulted at 37°C (Figure 2C). The *sec23-1*, *sec21-3* and *sec18-1* (NSF) mutants all blocked newly synthesized GFP-Gos1p in the ER at 37°C (Figure 2D). By contrast, *pUG-trs20-1* and *pUG-trs20-6* behaved like *pUG-TRS20* under these conditions, where newly synthesized GFP-Gos1p exited the ER producing a punctate Golgi-like pattern (Figure 2C). Therefore, trafficking of GFP-Gos1p from the ER to the Golgi is unaffected in the Trs20 mutants.

#### Transport to the vacuole

**Figure 3:**
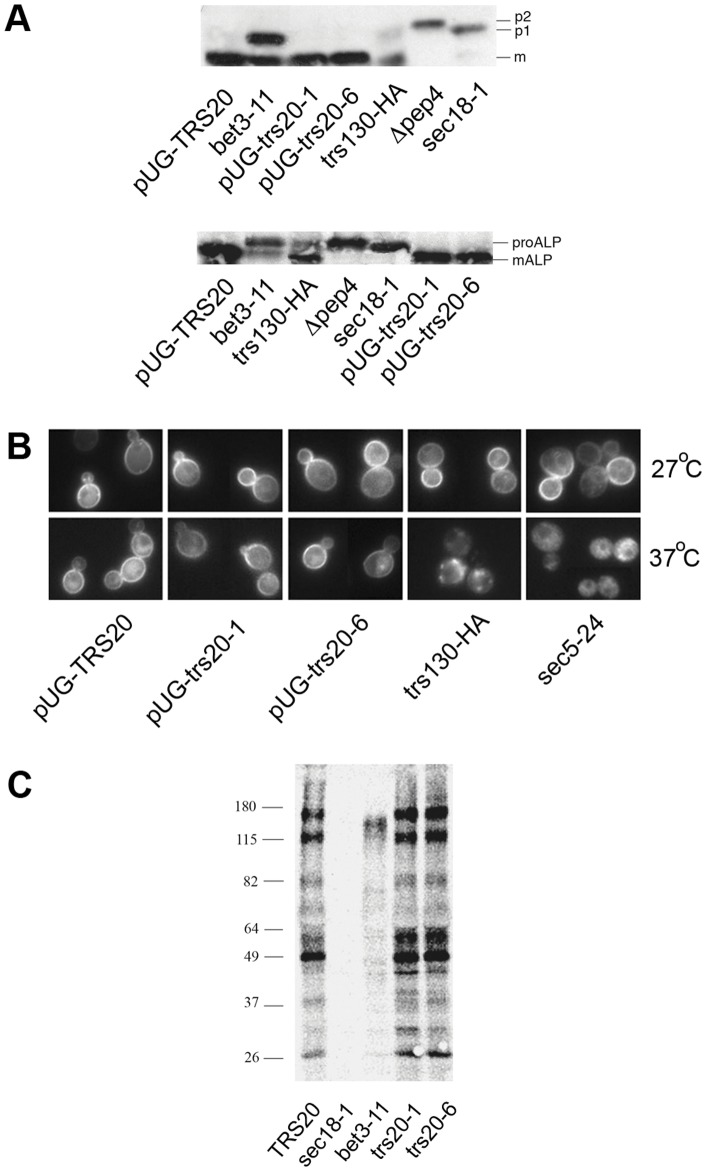
Transport of CPY, ALP and Gap1p in the Trs20 mutants. (**A**) CPY and ALP transport. Western blot analysis of CPY (top) and ALP (bottom) with total protein extracts from the indicated strains after 2 hours at 37°C. Mature, ER and Golgi forms of CPY are indicated by m, p1 and p2, respectively, and the ALP mature and precursor forms by mALP and proALP, respectively. (**B**) Fluorescence microscopy of the transmembrane protein GFP-Gap1(K9K16). Cells were grown in raffinose to log phase, galactose was added and the cells were then incubated at 27°C or at 37°C for 3 hours. (**C**) General secretion is not blocked in Trs20 mutants. Cells were grown to early log phase at 25°C, preincubated at 37°C for 30 minutes, radiolabeled for 15 minutes, and then chased for 30 min. The growth medium was separated from the cells by centrifugation, precipitated with TCA and separated by SDS-PAGE. Molecular weight markers (kDa) are indicated on the left.

The vacuolar hydrolase carboxypeptidase Y undergoes core glycosylation in the ER (p1 form), is modified to the p2 form in the Golgi and then sorted to the vacuole where it undergoes maturation to the mature (m) form [26]. Immunoblot analysis using an anti-CPY antibody on protein extracts from *bet3-11* and *sec18-1* mutant cells incubated at the restrictive temperature showed an accumulation of the p1 form, as expected [27], while the Δ*pep4* mutant (required for protease activation in the vacuole) [28] showed the presence of the p2 form (Figure 3A). The TRAPP II mutant *trs130-HA^ts^* accumulated some intermediate late forms of the CPY protein as previously described [13], [15]. By contrast, there was no trace of any intermediate CPY forms in the Trs20 mutants that showed a processing pattern identical to *pUG-TRS20* cells (Figure 3A).

Alkaline phosphatase is also modified en route to the vacuole but follows an alternative route to that taken by CPY [29]. ALP is transported through the secretory pathway as a 74 kDa precursor (proALP) that is cleaved in the vacuole to produce the mature 72 kDa form (mALP). The proALP protein could be observed in the *bet3-11*, *sec18-1*, *pep4*, and *trs130-HA^ts^* mutants but not in the Trs20 mutants (Figure 3A) that appeared to be identical to the *pUG-TRS20* cells where only the mature form of the enzyme was observed. These data indicate that there is no major defect in these transport pathways to the vacuole in *pUG-trs20-1* or *pUG-trs20-6*.

#### Transport to the plasma membrane

To test anterograde trafficking to the PM, we followed the transport of newly synthesized Gap1p, a transmembrane general-amino-acid permease, which is transported to the PM under nitrogen-limiting conditions but is ubiquitinated and targeted to the vacuole in nitrogen rich medium. Mutation of the ubiquitination sites in Gap1p (K9K16) leads to transport to the PM independently of the availability of nitrogen [30], [31]. The Trs20 mutants, a Trs130 ts mutant (CBV476, see Materials and Methods) and the exocyst mutant *sec5-24* [32] were transformed with a plasmid that expresses GFP-tagged Gap1 (K9K16) under the control of a galactose-inducible promoter; expression is repressed when cells are grown in glucose or raffinose and can be induced by the addition of galactose. Cells were grown in raffinose, then galactose was added and the cells were re-incubated at 27°C or shifted to 37°C for 3 hours. At 27°C all strains showed Gap1-GFP fluorescence at the PM. This was also found in the *pUG-TRS20* cells and Trs20 mutants at 37°C, but transport to the PM was blocked in the Trs130 mutant and the *sec5-24* mutant at the restrictive temperature (Figure 3B).

Finally, we analysed general secretion into the medium (see Materials and Methods). General secretion was blocked at the restrictive temperature in the *sec18-1* and *bet3-11* mutants but both *pUG-trs20-1* and *pUG-trs20-6* showed a pattern very similar to *pUG-TRS20* cells (Figure 3C).

Taken together, these results show that the Trs20 ts mutants do not have any major defect in a diverse range of anterograde trafficking pathways.

### Trs20 mutants are hypersensitive to calcofluor white

**Figure 4:**
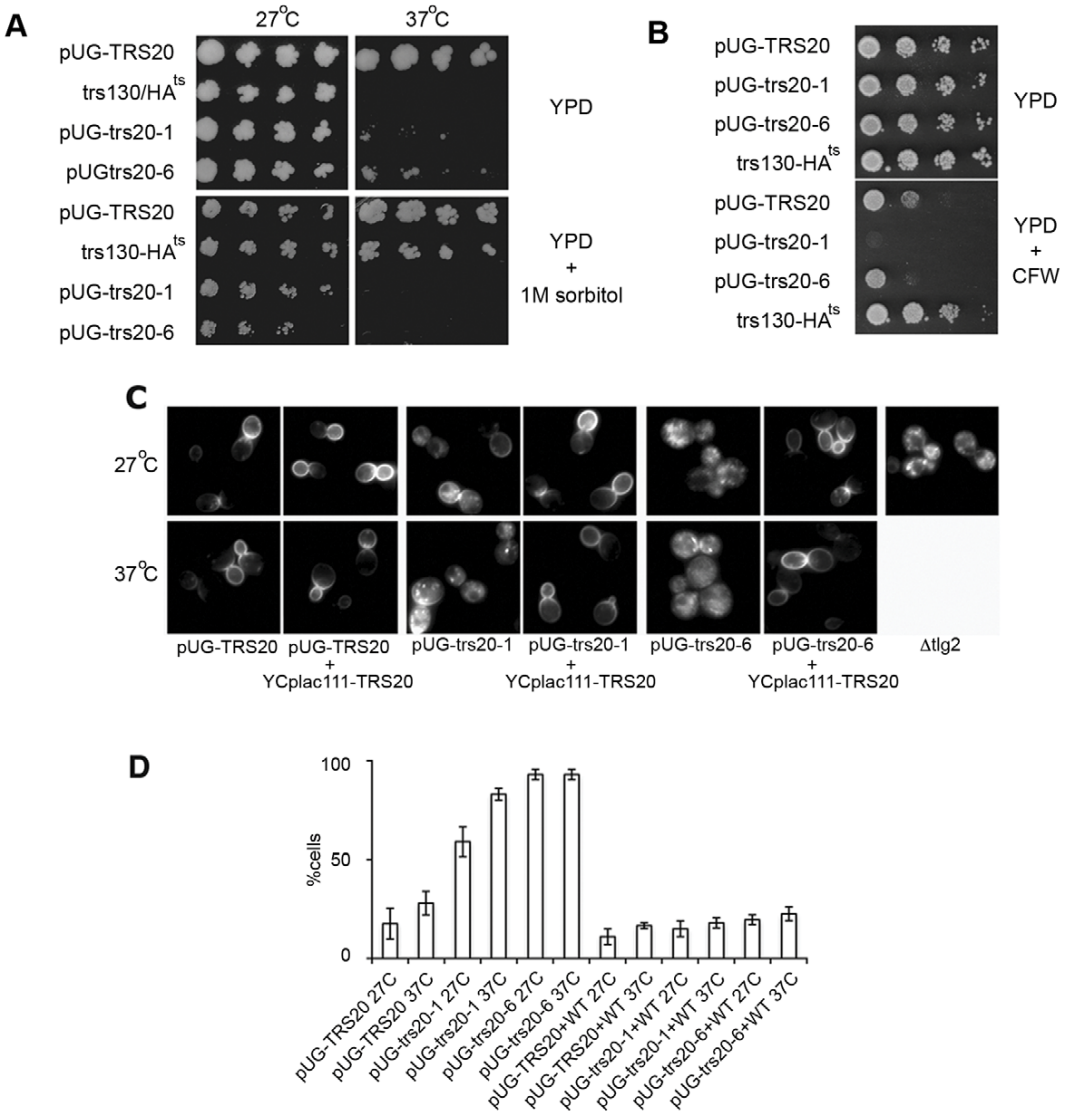
Trs20 mutants are hypersensitive to calcofluor white and mislocalize Snc1p. (**A**) *pUG-TRS20, pUG-trs20-1, pUG-trs20-6* and *trs130-HA^ts^* cells were serially diluted and replica plated on YPD or YPD + 1 M sorbitol and incubated at 27°C or 37°C for 3 days or (**B**) on YPD or YPD containing 10 μg/ml of calcoflour white (CFW) and incubated at 27°C for 3 days. (**C**) The pUG-trs20 mutants mislocalize Snc1p. Early log phase cells expressing GFP-Snc1p were grown at 27°C or shifted to 37°C for 30 minutes. GFP-Snc1p is constitutively mislocalized in the *pUG-trs20-6* mutant, similarly to Δtlg2 cells. The mislocalization can be rescued at 27°C and 37°C by co-expressing a WT copy of the Trs20 gene (YCplac111-TRS20). (**D**) A graph depicting the percentage of cells with mislocalized GFP-Snc1p, mean values ± SD, 3 replicates, n >100.

Mutation of the TRAPP II subunit Trs130, which has a role in transport from the Golgi to the plasma membrane, leads to a defect in cell wall integrity that is manifested as an osmotically-sensitive phenotype that can be rescued on osmotically-stabilized media [33]. In contrast to the *trs130-HA^ts^* mutant, growth of the Trs20 mutants at the restrictive temperature could not be rescued by growing the cells on 1 M sorbitol (Figure 4A) suggesting that they do not have such a cell wall integrity defect.

However, we found that the Trs20 mutants exhibit hypersensitivity to calcofluor white (CFW), a compound that is toxic for yeast cells. CFW binds primarily to chitin, a component of the yeast cell wall, and mutants with reduced chitin levels exhibit CFW resistance while increased chitin levels lead to hypersensitivity [34]. Trs130 mutants have been shown to be defective in the transport of the chitin-synthesizing enzyme Chs3p to the PM (13), which could account for their observed resistance to CFW (Figure 4B) [33]. By contrast, we found that the Trs20 mutants are hypersensitive to CFW at the permissive temperature of 27°C compared to *pUG-TRS20* cells (Figure 4B).

### Recycling of Snc1p is disrupted in the Trs20 mutants

CFW hypersensitivity could result from increased chitin synthesis or increased levels of chitin in the cell wall due to inefficient recycling of Chs3p from early endosomal-like structures (chitosomes) to the Golgi and thus increasing its level at the PM [34]. A number of endocytic mutants have been shown to have a CFW hypersensitive phenotype [35]. Considering this, and the absence of any obvious exocytic transport defects in the Trs20 mutants, we looked for a possible involvement in endocytic processes. Localization of the SNARE protein Snc1p has been developed as an assay for analysing trafficking defects from early endosomes to the Golgi [36]. Snc1p recycles from the plasma membrane via an early endosomal compartment to the Golgi from where it is transported back to the PM. Fluorescence microscopy of GFP-tagged Snc1p under steady state conditions in *pUG-TRS20* cells showed localization at the plasma membrane and polarized localization at the bud neck at 27°C and after shifting cells to 37°C (Figure 4C). The *pUG-trs20-1* mutant showed partial mislocalization of Snc1p even at the permissive temperature and this was exacerbated by a shift to the restrictive growth conditions of 37°C (Figure 4C, D). Strikingly, almost all of the *pUG-trs20-6* mutant cells showed a mislocalization of Snc1p to an internal diffuse or spotty pattern even at the permissive temperature (Figure 4C, D). This mislocalization of Snc1p could be rescued by co-expressing a WT version of the Trs20 gene (Figure 4C, D). Therefore, the Trs20 mutants are defective in GFP-Snc1p recycling suggesting that they play a role in an endocytic pathway, possibly from early endosomes to the Golgi. The constitutive mislocalization of Snc1p in the Trs20 mutants at the permissive temperature is similar to that observed in cells deleted for the Tlg2 gene (Figure 4C) [36], which encodes a SNARE protein involved in early endosome-to-Golgi trafficking [37]. However, it was also possible that the ts phenotype and the Snc1p recycling defect were separate phenomena, an issue that is addressed below.

### Genetic interactions of *pUG-trs20-1* and *pUG-trs20-6*

The data described above clearly show that the phenotypes we observe in the mutants are indeed due to defects in Trs20 function as they can be complemented by co-expression of the WT gene. However, as the Trs20 ts mutants contain a GFP tag and their expression is driven by the Met25 promoter, we sought to further corroborate these data by testing genetic interactions between the mutants and other genes that have been described as interacting with Trs20. In particular, given the Snc1p recycling defect, we tested genetic interactions between the Trs20 mutants and strains carrying a gene-deletion for the v-SNARE Tlg2 and the GTPase Arl3, which have been implicated in endocytic recycling [37], [38], with Apl5, a subunit of the AP-3 complex, and Nyv1, a SNARE involved in vacuolar fusion, which operate in late Golgi/post-Golgi events [39], [40]. Trs20 has been described as having synthetic growth defects with Arl3 [41], Tlg2 and Apl5 [42] mutants.

However, we found that homozygous *pUG-trs20-1* and *pUG-trs20-6* mutants do not sporulate and mutant spores dissected from a heterozygous diploid show poor viability (Figure S3A, B) compared to those expressing a WT copy of the gene (Figure. S3C). In addition, those spores that did grow gave rise to small colonies (Figure S3A, B) but further outgrowth of these clones at the permissive temperature was similar to *pUG-TRS20*. The poor viability may be due to an inability to overcome a post-germinative phase of growth since microscopic examination showed that at least 90% of the spores that did not give rise to visible colonies produced microcolonies of 8–16 cells (Figure S3D). This phenotype appears to be specific for the Trs20 mutants as spores deleted for the TRAPP subunit Trs85 (Δ*trs85*) or the *bet3-11* or *trs130-HA^ts^* mutants did not show such a phenotype (Figure S3E and data not shown).

The poor spore viability precluded the possibility of using a classical tetrad analysis approach to look for genetic interactions. We therefore adopted an alternative approach in which we generated doubly deleted strains (for Trs20 and the gene-of-interest) that co-expressed either the pUG23-trs20-1 or the pUG23-trs20-6 mutant from one plasmid and the WT version of Trs20 on another plasmid, with each plasmid containing a different selectable marker. We reasoned that if there were synthetic lethality between a Trs20 mutation and the gene-of-interest then these cells would be incapable of losing the WT Trs20 gene when cultured in non-selective medium. A full description of the procedure is given in Materials and Methods.

Both of the Trs20 mutants showed the same genetic interactions, a synthetic growth defect with *Δapl5* at 37°C but no interaction with *Δnyv1* (Figure S4A). More significantly, both of them were synthetically lethal with *Δtlg2* and *Δarl3* as judged by their inability to lose the WT copy of the gene under non-selective conditions (Figure S4B). Therefore, *pUG-trs20-1* and *pUG-trs20-6* show the same interactions reported in high-throughput genetic screens, supporting the view that the observed phenotypes are due to a dysfunctional Trs20 protein.

We then tested if expression of the small GTPase Ypt32p or the GTP-locked form of Ypt32p (ypt32Q72L) [43] from a low copy-number plasmid could rescue the ts phenotype of the Trs20 mutants. Overexpression of Ypt31/32p has been shown to rescue the deletion and ts phenotype of Trs130 mutants but only weakly rescue cells deleted for Trs120 [14], [15], which has a role in endocytic recycling [13]. Compared to the suppression of *trs130-HA^ts^*, overexpression of Ypt32p only weakly suppressed the ts phenotype of *pUG-trs20-1* (Figure S5). In repeated experiments, the WT version of Ypt32p rescued the ts phenotype of *pUG-trs20-1* better than ypt32Q72L, in contrast to the stronger suppressive effect of the GTP-locked form of Ypt32p on other TRAPP mutants [14], [15]. This difference was never observed for the *pUG-trs20-6* mutant that was very weakly suppressed by both versions of Ypt32p (Figure S5).

Trs130 appears to function together with Ypt31/32p and the phosphatidylinositol-4-kinase Pik1p in controlling transport processes at the late Golgi [15], [44]. We found that expression of Pik1p from a high copy number plasmid rescued the ts phenotype of *trs130-HA^ts^*, similarly to the expression of Ypt32p (Figure S5), but not that of the Trs20 mutants (data not shown). Together with previous studies [14], [15], [44] these results support the notion that the function of Ypt31/32p and the TRAPP II complex may be regulated by the lipid composition of the Golgi apparatus. However, the differences observed in the genetic interactions of *pUG-trs20-1, pUG-trs20-6* and *trs130-HA^ts^* and the differential phenotypic effects of these mutants described above would argue that Trs20p has a role in a process other than that involving Trs130p, Ypt31/32p and Pik1p.

### The temperature-sensitive phenotype is separable from other phenotypes caused by mutation of Trs20

The observation that Snc1p recycling is defective even at the permissive temperature suggested that this phenotype could be unlinked to the temperature sensitivity. To better define the effects of the Trs20 mutations and the observed phenotypes, the mutant ORFs were recloned under control of the Trs20 promoter and terminator in the plasmid YCplac111 (see Materials and Methods) to generate YCplac111-trs20-1 and YCplac111-trs20-6. These constructs were then introduced into the Δtrs20 background by plasmid swapping generating mutants *YCplac-trs20-1* and *YCplac-trs20-6*. We checked the effect of the *YCplac-trs20-6* mutant on a number of trafficking pathways and, as for the GFP tagged mutant, no effect was observed on Rer1p (Figure S6A), Gos1p (Figure S6A, B) or Gap1p (Figure S6C) transport.

**Figure 5:**
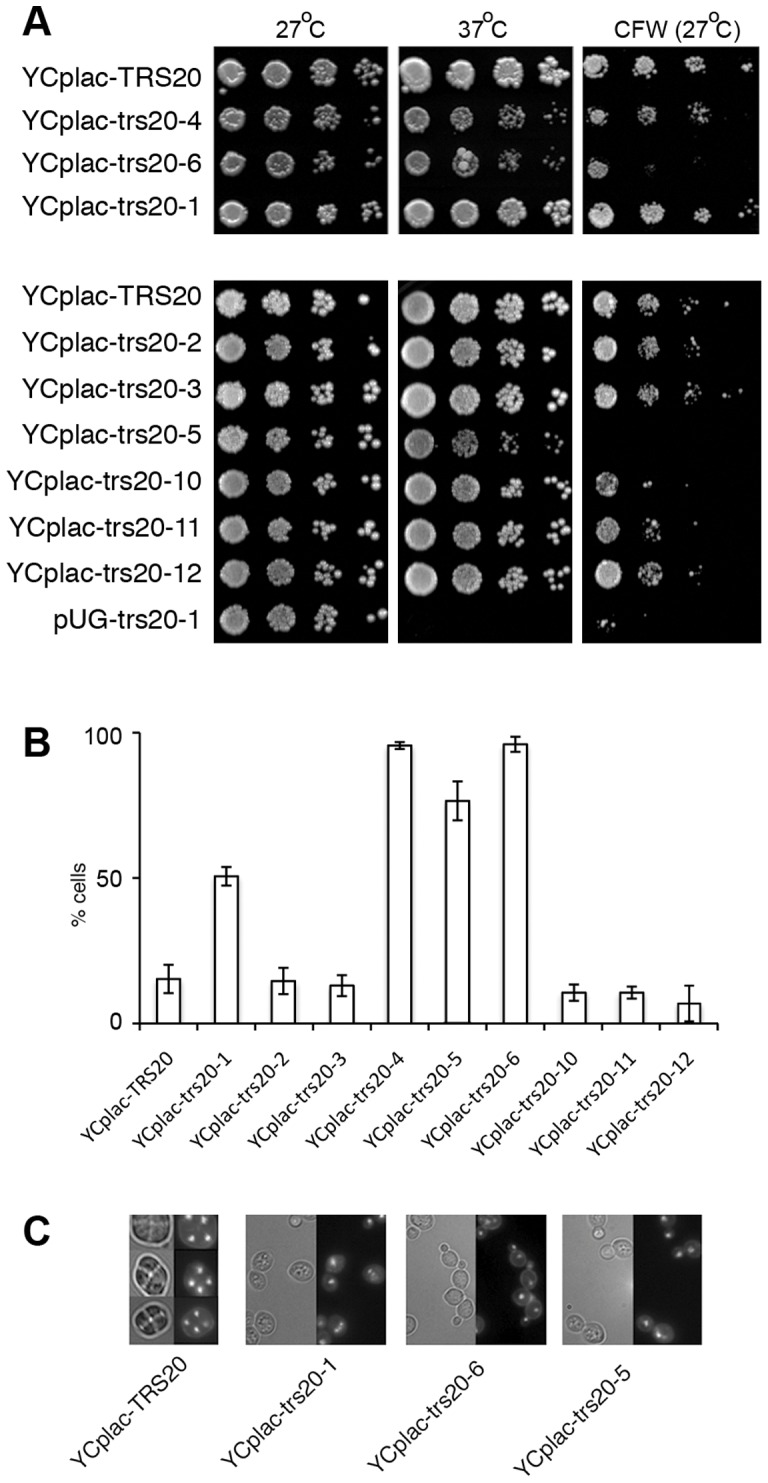
Phenotypes of *YCplac-trs20* mutants. (**A**) Serial dilutions were spotted onto YPD plates or onto YPD containing 10 μg/ml of calcoflour white (CFW) and incubated for 3 days at the indicated temperatures. (**B**) Localization of GFP-Snc1p in the indicated strains. A graph depicting the percentage of cells with mislocalized GFP-Snc1p, mean values ± SD, 3 replicates, n >100. (**C**) *YCplac-trs20* mutants do not undergo sporulation or meiosis. Light microscopy (left) and Hoescht staining (right) in each panel. In cells expressing a WT copy of Trs20, asci and the four products of meiosis are readily identified. No asci or meiosis were observed in the Trs20 mutants in sporulating cultures where the mother and daughter cell typically contain a single nucleus.

The *YCplac-trs20-1* mutant did not exhibit the ts phenotype or hypersensitivity to CFW that was observed in *pUG-trs20-1* while the partial Snc1p recycling defect at 27°C was similar (Figure 5A, B). *YCplac-trs20-6* had a weak ts phenotype but retained the CFW hypersensitivity (Figure 5A), and Snc1p was still completely mislocalized (Figure 5B). However, both mutant constructs were still incapable of supporting sporulation, a defect that could be rescued by co-expression of WT Trs20 (data not shown). Hoechst staining of sporulating cultures showed that this is due to a block in meiosis (Figure 5C), although it should be noted that this staining does not distinguish between a defect in DNA synthesis and chromosome segregation.

**Figure 6:**
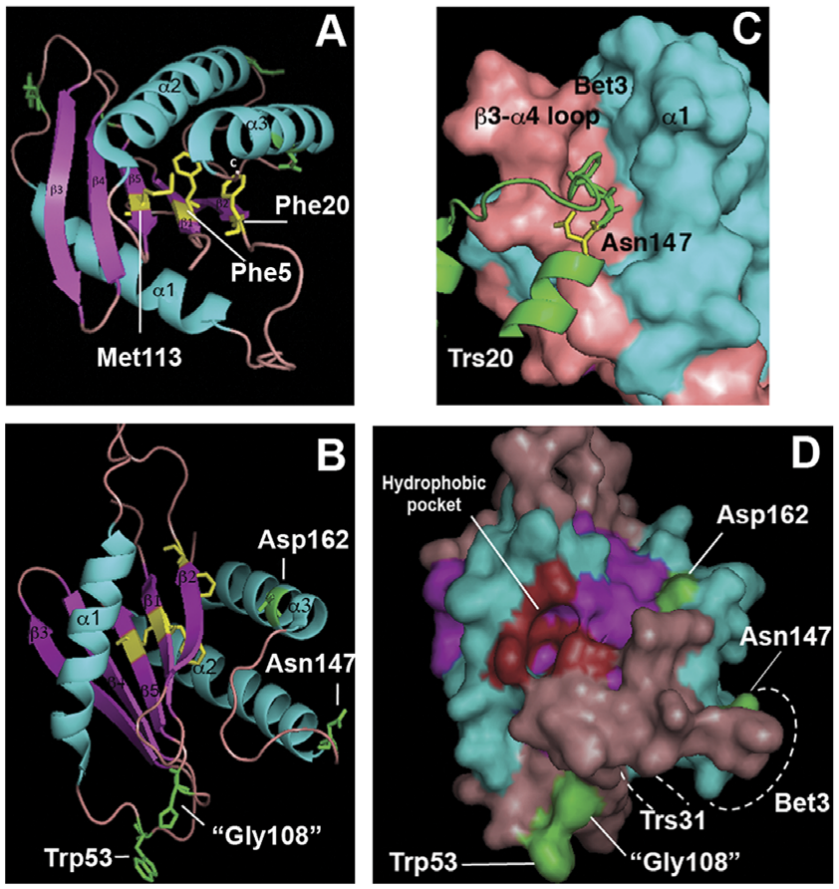
Location of the studied mutations in the three-dimensional model of sedlin. The model is based on the published structure of mouse sedlin [19], but amino acid numbers refer to the yeast Trs20 protein. (**A**) Location of mutated residues (yellow) in the hydrophobic core. (**B**) Location of mutated surface-exposed residues (green). The side chains of the glycine 108 residue (“G108”) are shown as the histidine residue in sedlin to mimic the tryptophan substitution in the G108W mutant (see text). (**C**) Detailed view showing the Asn147 residue of Trs20 (yellow) within the hydrophobic pocket of Bet3 [11]. (**D**) Surface rendition of the structure in (B). The residues contributing to the formation of the hydrophobic pocket [19] are shown in red. Dashed lines indicate the occupancy of the hydrophobic groove of Bet3 by Trs20 and of the groove A of Trs20 by Trs31. Visualization of the 3-D structure (accession number 1H3Q) was done using the MacPyMOL program.

We created additional single and multiple mutation combinations to try to understand the contribution of the mutated residues to the observed phenotypic defects (see Figure 5A, B and Table 1 for the phenotypes of the mutants described below). The mutations in the *YCplac-trs20-1* mutant, which shows only a defect in sporulation and partial Snc1p mislocalization, are within the hydrophobic core (F5S) or exposed on the surface (W53R) (Figure 6A, B). The W53 residue has been suggested to be located within a protein interaction region [19]. However, even when W53R was expressed in combination with other mutations such as N147G or D162Y (see below), no phenotypic defects were observed (mutants *YCplac-trs20-2* and *YCplac-trs20-3*). In particular, these mutants were capable of supporting sporulation and undergoing meiosis (data not shown) suggesting that the F5S mutation in *YCplac-trs20-1* might have been responsible for the sporulation defect, but we found that the single point mutation F5S (*YCplac-trs20-10*) was capable of sporulation. Reversion of the F20S mutation of *YCplac-trs20-6* to WT (mutant *YCplac-trs20-5*) showed only a partial recovery of Snc1p mislocalization but still showed CFW hypersensitivity and failed to sporulate (Figure 5B, C). The G108W and M113K mutations, therefore, seem to be sufficient to account largely for the phenotypes observed. Neither of the single mutants M113K (*YCplac-trs20-11*) nor G108W (*YCplac-trs20-12*) produced any defective phenotypes. Curiously, combining the W53R mutation with G108W and M113K (*YCplac-trs20-4*) worsened the Snc1p mislocalization defect but recovered somewhat the CFW sensitivity. Finally, while mutation of the highly-conserved surface-exposed residues N147G and D162Y had no observable effect, (in combination with the W53R mutation, *YCplac-trs20-2* and *YCplac-trs20-3*, respectively) they resulted in lethality when combined with the G108W and M113K mutations (mutants *YCplac-trs20-7* and *YCplac-trs20-9*).

**Table 1:** Summary of phenotypes of Trs20 mutants.

Strain	Mutations	Viaility	Ts	CFW	SPOR.	SNC1
*YCplac-TRS20*	WT	+	-	+	+	-
*pUG-TRS20*	WT	+	-	+	+	-
*pUG-trs20-1*	F5S:W53R	+	+++	++++	-	+
*YCplac-trs20-1*	F5S:W53R	+	-	+	-	+
*YCplac-trs20-2*	W53R:N147G	+	-	+	+	-
*YCplac-trs20-3*	W53R:D162Y	+	-	+	+	-
*YCplac-trs20-4*	W53R: G108W: M113K	+	+	++	-	+++
*YCplac-trs20-5*	G108W:M113K	+	+	++++	-	++
*pUG-trs20-6*	F20S:G108W: M113K	+	+++	++++	-	+++
*YCplac-trs20-6*	F20S:G108W: M113K	+	+	+++	-	+++
*YCplac-trs20-7*	G108W:M113K: D162Y	Inviable	na	na	na	na
*YCplac-trs20-8*	F5S:G108W: M113K	Inviable	na	na	na	na
*YCplac-trs20-9*	G108W:M113K: N147G	Inviable	na	na	na	na
*YCplac-trs* *20-10*	F5S	+	-	++	+	-
*YCplac-trs* *20-11*	M113K	+	-	+	+	-
*YCplac-trs* *20-12*	G108W	+	-	+	+	-

ts, temperature sensitivity: – none, + weak, +++ strong; CFW, calcofluor white sensitivity: + weak to ++++ hypersensitive; SPOR, sporulation: + yes, – no; SNC1, Snc1p localization: – no to +++ strong mislocalization; na, not applicable.

How might we interpret the effects of the mutations that are seen only when more than one site is mutated? The only mutated residue that has been shown to interact directly with another protein is N147. This residue forms part of an interaction surface that fits into the hydrophobic pocket of Bet3 (Figure 6C, D) [11]. Mutation to glycine (N147G) would remove the hydrogen bonding between asparagine and the Bet3 molecule that on its own may only weaken but not abolish the interaction. The substitution of the hydrophobic methionine residue by the negatively charged lysine in *YCplac-trs20-9*, which is located in the hydrophobic core, could cause a conformational change that exacerbates this weakened interaction with Bet3, resulting in lethality. The lethality with the surface exposed D162Y mutation, which would not impinge on Bet3 binding (Figure 6D) [11], may be due to similar considerations with another, so far unknown, binding partner.

A small glycine residue at position 108 is substituted by a large tryptophan residue in G108W. The equivalent residue is a histidine (H80) in the human sedlin protein (represented in Figure 6B and D to mimic the tryptophan substitution. This residue is directed away from groove A where Trs31 interacts, Figure 6D). This is in close proximity to the W53 site and the effects seen with mutants mutated for either of these residues may have the same underlying consequences, at least partially as the F5S:W53R and G108W:M113K mutants differ in their sensitivity to calcofluor white. Indeed, the F5S and M113K mutations, which are associated with W53R and G108W, respectively, are in close proximity in the hydrophobic core (Figure 6A) and combining the G108W and M113K mutations with the F5S mutation resulted in inviability (mutant *YCplac-trs20-8*). Mutation of these residues in the hydrophobic core may cause a conformational change that affects the interaction of surface exposed residues with binding partner proteins. Alternatively, as the F5 residue is adjacent to a residue (Ala 6 in Trs20, Val 8 in sedlin) [19] that forms part of the hydrophobic pocket in sedlin (Figure 6D), which is a putative protein interaction domain [19], the F5S may affect interaction with a protein that binds both within the hydrophobic pocket and with the exposed W53 residue. The substitution of glycine 108 with tryptophan may result in stereochemical hindrance in binding to this putative protein partner, therefore acting with the M113K mutation, also within the hydrophobic pocket, like the F5S:W53R mutant combination.

## Discussion

Notwithstanding more than 12 years since the discovery of the TRAPP complex [45], there is little evidence regarding the function of the Trs20 subunit, partly due to Trs20 being an essential gene. To address this problem we undertook an unbiased mutagenic strategy to isolate temperature-sensitive mutants of Trs20, as classically these have been informative for analyzing the function of essential genes. In the initial mutagenic screen we could only identify ts mutants after mutagenizing a GFP-tagged version of Trs20. Numerous lines of evidence show that these tagged mutants represent *bone fide* conditional mutants of Trs20: (i) the GFP-tagged WT protein does not show any of the phenotypes associated with the mutant proteins, (ii) the tagged mutant proteins rescue the lethality caused by deletion of the Trs20 gene, (iii) co-expression of the WT Trs20 gene rescues all of the mutant phenotypes, (iv) the mutants show the same genetic interactions described in high-throughput genetic interaction screens, and (v) the untagged mutant versions show a subset of the phenotypes of the tagged mutants. The fact that the ts phenotype was found in the GFP-tagged mutants, but not in the untagged mutants, can be explained by a misfolding of the tagged protein, or steric hindrance in its interaction with other proteins, that occurs only at the restrictive temperature in the presence of the mutations.

The constitutive mislocalization of Snc1p and CFW hypersensitivity observed in the untagged mutants suggest that these represent functions distinct from those affected by the ts phenotype. Both of these phenotypes could result from a defect in endosome-to-Golgi trafficking or recycling to the PM since Snc1p recycles from the plasma membrane to the Golgi via an early endosomal compartment [36] and Chs3p, a cell surface enzyme required for chitin synthesis, follows a recycling pathway from early endosomal-like structures to the PM [34]. Indeed, synthetic-lethality was observed between the Trs20 mutants and Δtlg2, a SNARE protein involved in early endosome-to-Golgi transport [37], [46]. They also showed a synthetic lethal phenotype in combination with Δarl3, a small GTPase that is required for the recruitment of another GTPase, Arl1p, to the late Golgi. Arl1p, in turn, can directly bind and recruit GRIP domain proteins, which are required for endosome-to-Golgi traffic [47], [48], [49]. In addition, Δarl3 cells show synthetic lethality when combined with mutations in other proteins that affect recycling to the Golgi such as Tlg2, Ypt6, Ric1 and Gyp1 [50], [51]. We found that some homozygous diploid Trs20 mutants are unable to sporulate or to undergo meiosis and it has been shown that retrograde trafficking from the endosome is essential for sporulation [52]. It is unclear to what extent the effects of the Trs20 mutants on these two processes are linked, however, as *YCplac-trs20-1* shows only a partial defect in Snc1p localization yet is completely blocked in sporulation (Table 1). Interestingly, Trs85 was first identified as a sporulation-deficient mutant that had a defect in meiosis [53]. Trs20 mutant phenotypes differ from Δtrs85, however, with respect to post-germinative spore growth (Figure S3).

While Trs20p has been reported to link the TRAPP I complex to a TRAPP II-specific component, possibly Trs130p [18], we found that in contrast to the calcofluor white resistance shown by *trs130-HA^t^* ^s^ and the rescue of the ts phenotype by sorbitol, Trs20 mutants show hypersensitivity to CFW and the ts phenotype cannot be rescued by sorbitol. Additionally, the suppression of *trs130-HA^ts^* by Ypt32p and Pik1p overexpression together with the defects in the transport of CPY and ALP to the vacuole and Gap1p transport to the PM in *trs130-HA^ts^* but not in Trs20 mutants suggest that Trs20p and Trs130p operate in different pathways. On the other hand, Sedlin, the mammalian orthologue of Trs20p, has been reported to interact directly with the TRAPP II subunit TRAPPC9 (yeast Trs120p) [54]. Trs120p appears to function in early endosome-to-Golgi transport [13], so Trs20p might operate together with Trs120p in this pathway.

The ts phenotype of the tagged mutants could reflect the essential function of Trs20 although we did not find any other phenotypes related to the temperature sensitivity. If this essential function is as part of the TRAPP I complex in ER-to-Golgi trafficking, we speculate that it should mediate the transport of a specific cargo(es) as no defects were found in the transport of a number of reporter molecules to the Golgi, vacuole, plasma membrane or in general secretion. This hypothesis is currently under investigation.

Trs20/sedlin is a longin-domain protein that has been proposed to exert a regulatory function through multiple protein-protein interactions [19]. The crystal structure of the TRAPP complex subunits showed that two adjacent regions of sedlin contact the TRAPP subunits Trs31 and Bet3 [11]. The Bet3 interface of sedlin contains the surface-exposed N147G mutation studied here that by itself was phenotypically neutral but in combination with mutations in ß5 resulted in lethality, possibly through a loss of contact with Bet3. Similarly, the surface-exposed D162Y mutation in α3 by itself was phenotypically neutral but in combination with mutations in β5 resulted in lethality. This may represent an interaction domain for another Trs20 binding partner. Finally, the hydrophobic pocket together with the surface exposed W53 residue may represent an interaction surface for a protein that is required for sporulation. The mutational analyses described here support the suggestion that Trs20/sedlin has a role in diverse functions in the cell by acting as an adapter protein via multiple protein-protein interactions.

## Materials and Methods

### Strains, plasmids and growth conditions

Strains and plasmids used in this study are listed in Table S1 and Table S2. All endogenous gene deletions are disrupted with the kanamycin resistance gene (KanR) in the BY4743 background (obtained from EUROSCARF). Strains VSY446 (trs130-HAts::HIS3MX6), DTY159 (*sec5-24*), RSY271 (*sec18-1*), and RSY427 (*sec23-1*) have been described previously [15], [32], [55]. VSY446 is suc2-Δ9 and grows poorly with raffinose as a carbon source. To study the effect of the Trs130 ts mutation on Gap1-GFP transport, strain CBV474 was generated by mating VSY446 with CWY474 (a spore clone derived from BY4743 with the opposite mating type, this study), sporulating the diploid and selecting spore clones that segregated 2∶2 for histidine prototrophy that were ts (diagnostic for the presence of the trs130 ts mutation) and showed robust growth with raffinose as a carbon source (diagnostic for the SUC2 gene). CBV474 cells were then transformed with the GFP-tagged Gap1-expressing plasmid and analyzed as described below. Strain sec21-3 was obtained by transforming the diploid strain Δsec21/SEC21 (KanR/kanS) with plasmid p315-sec21-3 (LEU2) [25], sporulating, and selecting spores after tetrad analysis that were KanR, LEU2, ts. The plasmid pUG23-Trs20 was constructed by PCR amplification of the ORF (open reading frame) from pYCG-YBR254c (the Trs20 cognate clone from EUROSCARF) and cloning in plasmid pUG23 (CEN, HIS3). This plasmid was used for error-prone PCR (see mutagenesis) to generate the ts mutant constructs pUG23-trs20-1 and pUG23-trs20-6. The Trs20 insert in pYCG-YBR254c, containing the upstream 292 nucleotides and downstream 252 nucleotides, was recloned *Hin*dIII/*Xba*I into the plasmid YCplac111 (CEN, LEU2) to generate YCplac111-TRS20. The Trs20 mutations were substituted into YCplac111-TRS20 by subcloning, site-directed mutagenesis and megaprimer PCR [56]. The plasmids YCplac111-Bet3 and YCplac33-Bet3 were constructed by amplifying a genomic fragment that contained the upstream 531 and downstream 106 nucleotides of Bet3 and cloned in the vectors YCplac111 (CEN, LEU2) and YCplac33 (CEN, URA3), respectively. The Bet3 ts mutant *bet3-11* was generated by site-directed mutagenesis using YCplac111-Bet3 as template (see mutagenesis). The plasmid expressing pUG36-GOS1 was constructed by amplifying the ORF from genomic DNA and cloning in the vector pUG36 (CEN, URA3). The correct identity of all constructs was verified by sequencing. Plasmids pUG36-YPT32 and pUG36-ypt32Q72L have been described previously [43], [57]. The Pik1 ORF was cloned with an N-terminal FLAG-tag in plasmid pESC-URA (2-micron, URA3, Stratagene).

For repressing and inducing expression of GFP-tagged proteins from pUG vectors, cells were grown to log phase in selective medium containing 1 mM methionine to repress expression, then washed and resuspended in methionine-free medium to induce expression. Cells were visualized 30 min after the induction. For GFP-tagged Gap1 under control of the galactose promoter, cells were grown to log phase in selective medium containing raffinose and induction was performed by adding galactose to 2% and continuing the incubation for 3 hrs before visualization.

To test for temperature-sensitivity on solid medium, cells were grown first in liquid medium to late log phase, each culture was diluted to an OD_600_ of 0.7, then serially diluted and replica plated onto solid YPD medium and incubated at the permissive or restrictive temperature as indicated in the text and Figure legends. A similar procedure was adopted to test suppression of the ts phenotype by sorbitol (YPD + 1 M sorbitol) or sensitivity to calcofluor white (YPD + 10 μg/ml Fluorescent Brightener 28, Sigma).

### Mutagenesis

Generation of Trs20 ts mutants using error-prone PCR: The plasmid pUG23-Trs20 (Met promoter, Trs20-GFP, CEN, HIS) was used as a template for error-prone PCR using primers against the methionine promoter region and the GFP tag (500 nM each primer). The amplification mix contained 80 μM dATP, 80 μM dGTP, 400 μM dCTP. 400 μM dTTP, 1.5 mM MgCl_2_, 0.3 mM MnCl_2_ and Taq DNA polymerase (EuroTaq) and amplification was performed using the following parameters: 94°C 5 minutes, 94°C 45 seconds, 55°C 30 seconds, 72°C 1 min, 72°C 10 min, 35 cycles. The resulting PCR product was purified by gel electrophoresis. The pUG23-Trs20 plasmid was digested with EcoRI and SalI to remove the Trs20 coding sequence to produce a gapped (linearized) acceptor plasmid that was purified by gel electrophoresis. Yeast cells deleted for the genomic copy of Trs20 (Δtrs20) containing the plasmid pYCG-YBR254c (Trs20, CEN, URA) were transformed with equimolar amounts of the gapped plasmid and mutagenized DNA. Recombination between the gapped plasmid and mutagenized DNA via the homologous recombinogenic ends of the DNA, not present in pYCG-YBR254c, reconstituted the pUG23-Trs20 plasmid with random mutations in the Trs20 gene. Cells were selected on plates without histidine and uracil, and then counterselected on plates containing 5-fluoroorotic acid (5-FOA, which selects against URA3-containing plasmids). Colonies that were +His, -Ura were tested at 27°C and 37°C, and temperature-sensitive clones selected. The plasmids were recovered by plasmid rescue, sequenced and re-transformed into Δtrs20 cells for further analysis. The N147G mutation was identified during the initial mutagenic screen while the D162Y mutation was generated by site-directed mutagenesis. These mutations were combined with the other mutations described in the text and Table 1 by subcloning.

The Bet3 mutant was generated by site-directed mutagenesis using the Stratagene QuikChange kit and the YCplac111-Bet3 construct as template. All introduced mutations were verified by sequencing. Initially two separate mutants were generated (G64A and A82L) corresponding to two previously described mutated sites [27], [58], [59]. Neither of the single mutations produced a ts phenotype (data not shown). We therefore combined both mutations by subcloning (replacing a *Sca*I/*Eco*RI fragment in the A82L mutant construct with the corresponding fragment from the G64A mutant construct) and the resulting double mutant plasmid (called *bet3-11*) was used to transform the diploid strain Δbet3/Bet3, followed by sporulation, tetrad analysis, and spore selection (KanR, LEU2).

### Analysis of synthetic genetic interactions

The following approach was used to search for synthetic genetic interactions. Cells deleted for the gene of interest (Δapl5, Δtlg2, Δarl3, Δnyv, each gene replaced with a kanamycin gene, KanR) were transformed with YCplac111-TRS20 (LEU2) and mated with the *pUG-trs20-1* and *pUG-trs20-6* mutants (Δtrs20, KanR, HIS3, expressing the ts Trs20 mutants from plasmid pUG23). Diploids were selected on double selective medium and then sporulated. The spores were dissected and the spore clones tested for kanamycin resistance and the presence of both selectable markers (LEU2, HIS3). Spore clones that were positive for all three markers were checked by PCR to verify the presence of both deleted genes (Δtrs20 and the gene-of-interest). Spores clones containing no gene deletion (but with both plasmids) were also taken as controls. The spore clones were inoculated into selective medium containing leucine but without histidine, grown overnight, re-inoculated into the same medium overnight, and then plated for single colonies on solid selective medium containing leucine but without histidine. Individual colonies were picked and streaked on solid selective medium containing either only histidine or only leucine and scored for retention of the YCplac111-TRS20 plasmid. Cells that contained a ts Trs20 allele plus a deletion of the gene-of-interest that were incapable of losing the wild type copy of the gene were scored as synthetic lethal.

### General secretion

Cells were grown in YPD at 23°C overnight, re-inoculated in fresh medium at an OD_600_ of 0.15, and incubated at 23°C to an OD_600_ of 0.4. The cells (2 OD) were centrifuged and then incubated in minimal medium for 20 minutes at 37°C, centrifuged and re-suspended in minimal medium minus methionine for 10 minutes at 37°C. After the addition of 100μCi radiolabeled Trans-35S label mix the cells were incubated for 15 minutes at 37°C and then chased with an excess of unlabeled methionine/cysteine containing 500 μg/ml of bovine serum albumin for 15 minutes, 37°C. After adding NaN_3_ and NaF (500 μM) and centrifugation, the growth medium was separated from the cells by centrifugation (1 minute, 4°C), 21 μl 100% TCA was added to 329 μl of the supernatant followed by incubation on ice for 60 minutes. After centrifugation, the pellets were washed twice with cold acetone, resuspended in sample buffer, separated by SDS-PAGE and analyzed by autofluorography.

### CPY and ALP Western blot analysis

Cells were grown in YPD at 23°C overnight, re-inoculated in fresh medium at an OD_600_ of 0.25, grown for a further 4 hours at 23°C and then shifted to 37°C for 2 hours. The cells were centrifuged, washed, and crude protein extracts were prepared by suspending the cell pellets in 200 μl of 2 M NaOH, adding 40 μl of 50% TCA and incubating on ice for 10 minutes. After centrifugation the pellets were resuspended in 100 μl of SDS-loading buffer and the proteins (15 μl aliquots) were separated by SDS-PAGE and subjected to Western blot analysis using an anti-CPY antibody (CHEMICON International, Inc) or an anti-ALP antibody (Invitrogen).

### Sporulation and Hoechst staining

Stationary phase cell cultures were washed and resuspended in sporulation medium (1% potassium acetate, 0.1% yeast extract, 0.05% glucose) and incubated at 27°C for 4 days. Sporulating culture aliquots (100 µl) were washed with 1 x PBS, incubated in 70% ethanol for 30 minutes, washed twice with 1 x PBS, and resuspended in 100 µl 1 x PBS with 2.0 µg/ml Hoechst for 5 min. The cells were centrifuged, resuspended in 10 µl 1 x PBS and observed by fluorescence microscopy.

### Fluorescence microscopy

Strains expressing GFP-tagged proteins were grown to log phase in minimal media and observed directly. Cells were viewed using a Nikon Eclipse TE2000-U microscope equipped with HBO 100 W UV lamp (OSRAM), cooled color camera DS-5Mc-U1 (Crisel Instruments), high-speed filter wheel system (Crisel Instruments) controlled by the MetaMorph software (MetaImage Series 7.0, Molecular Devices). Image processing was performed with Photo-Shop 8.0.1 (Adobe Systems).

## Supporting Information

Figure S1 (A) Fluorescence microscopy of cells expressing Trs20-GFP (*pUG-TRS20*, see Table S1). No specific localization pattern could be observed under different conditions. (27°C, 37°C, a shift from glucose to galactose containing media or from media containing 1 mM methionine to media without methionine). Flourescence microscopy (left) and light microscopy (right) in each panel. (B) Fluorescence microscopy of cells expressing the TRAPP subunit Bet3 tagged with GFP (Δbet3 + pUG23-Bet3) shows the typical punctate Golgi pattern.
(TIF)

Figure S2 **Western analysis of** ***pUG-trs20-1*** **or** ***pUG-trs20-6*** **mutants.** Δtrs20 cells transformed with the plasmids pUG23-Trs20, pUG23-trs20-1 or pUG23-trs20-6 were taken after incubation at the permissive temperature (27°C, 0) or after 1 or 2 hrs at 37°C, total protein extracts were separated by SDS-PAGE, blotted and immunodetected with an anti-GFP antibody. A protein extract from non-transformed BY4741 cells was run in parallel. * marks a non-specific band.
(TIF)

Figure S3. ***pUG-trs20-1*** **and** ***pUG-trs20-6*** **spores show poor growth following germination.** Heterozygous Trs20/Δtrs20 diploids carrying (A) the pUG23-trs20-1 or (B) the pUG23-trs20-6 plasmid were sporulated and subjected to tetrad analysis. The circled spore clones were Kan+, His+, all others were kan- (the Trs20 gene is deleted by replacement with a kanamycin resistance gene and the pUG23 plasmid carries a histidine selectable marker). The plates were incubated for 4 days at 27°C. (C) Tetrad analysis of Trs20/Δtrs20 diploids expressing a plasmid-borne wild type copy (pUG23-Trs20) of the Trs20 gene. The plates were incubated for 2 days at 27°C. (D) The defect is not due to a problem in germination. The spores that did not give rise to visible colonies were checked under the microscope and approximately 90% of them gave rise to microcolonies (left panel). Dissection of these microcolonies showed that the spores had undergone 3–5 replications before arresting growth (right panel). (E) Tetrad analysis of Trs85/Δtrs85 diploids. The circled spore clones were Kan+ (the Trs85 gene is deleted by replacement with a kanamycin resistance gene).
(TIF)

Figure S4 **Genetic interactions of** ***pUG-trs20-1*** **and** ***pUG-trs20-6*** **.** (A) Serial dilutions of *pUG-TRS20, pUG-trs20-1, pUG-trs20-6* and the indicated double mutant cells replica plated on YPD were incubated at the indicated temperature for 3 days. No further growth of the Trs20 mutants combined with Δapl5 was observed even after longer incubation times at 37°C (data not shown). (B) Synthetic lethal interaction of *pUG-trs20-1* and *pUG-trs20-6* with *Δtlg2* and *Δarl3*. Double mutants of *pUG-trs20-1* or *pUG-trs20-6* with the gene-of-interest were generated by mating and tetrad analysis (see Materials and Methods for details). All clones contained the mutated Trs20 gene on a plasmid with a HIS selectable marker and the WT gene on a plasmid with a LEU selectable marker. Following growth in medium containing leucine, individual colonies were tested for retention of the LEU-containing plasmid carrying the WT Trs20 gene, YCplac111-TRS20 (% LEU, see Materials and Methods for details). *The genotypes were verified by PCR. † Meiotic products from the same tetrad. The genotype of the *TRS20-TLG2* cells was inferred from the kanamycin sensitive phenotype (deleted genes carry a Kan resistance gene). Three additional kanamycin-sensitive clones tested showed approximately 40% retention of the LEU selectable marker (data not shown).
(TIF)

Figure S5 **Suppression analysis of** ***pUG-trs20-1*** **,** ***pUG-trs20-6*** **and** ***trs130-HA^ts^*** **cells.** Cells transformed with low copy number plasmids carrying pUG36-Ypt32 or pUG36-ypt32Q72L (Ypt32(Q)) were serially diluted, replica plated on YPD and incubated at the indicated temperature for 3 days. Pik1p, expressed from a high copy number plasmid, suppresses the temperature-sensitive phenotype of *trs130-HA^ts^* cells.
(TIF)

Figure S6 **The** ***YCplac-trs20-6*** **mutant does not affect Rer1p, Gos1p or Gap1p trafficking.** (A) Rer1p-GFP and Gos1p-GFP under steady state conditions (27°C). (B) Induction of Gos1p-GFP expression by shifting cells from medium containing 1 mM methionine to medium without methionine. Left panels, fluorescent microscopy, right panels DIC images. (C) Induction of Gap1p-GFP expression by shifting cells from raffinose to galactose. Left panels, fluorescent microscopy, right panels DIC images. Experiments were performed as described in Materials and Methods, see main text and Figures 2, 3.
(TIF)

Table S1 **Strains used in this study.**
(DOC)

Table S2 **Plasmids used in this study.**
(DOC)
