# Correction: Mutational Analysis of the Yeast TRAPP Subunit Trs20p Identifies Roles in Endocytic Recycling and Sporulation

**DOI:** 10.1371/annotation/118aea6f-8edc-4107-8012-29110a55a9b1

**Published:** 2013-05-02

**Authors:** Hichem Mahfouz, Antonella Ragnini-Wilson, Rossella Venditti, Maria Antonietta De Matteis, Cathal Wilson

There was an error in Funding statement. The correct version of the Funding is:

This study was supported by the Telethon grants GGP07075 to CW, GGP08143 to ARW, GGP06166 to MADM and the AIRC grant IG 8623 to MADM (http://www.telethon.it/). The funders had no role in study design, data collection and analysis, decision to publish, or preparation of the manuscript. 

