# Supplementary figures and images for: Mutational Analysis of the Yeast TRAPP Subunit Trs20p Identifies Roles in Endocytic Recycling and Sporulation
Source: PLoS One. 2012 Sep 26;7(9):e41408. doi: 10.1371/journal.pone.0041408 (PMC3458868; doi:10.1371/journal.pone.0041408)

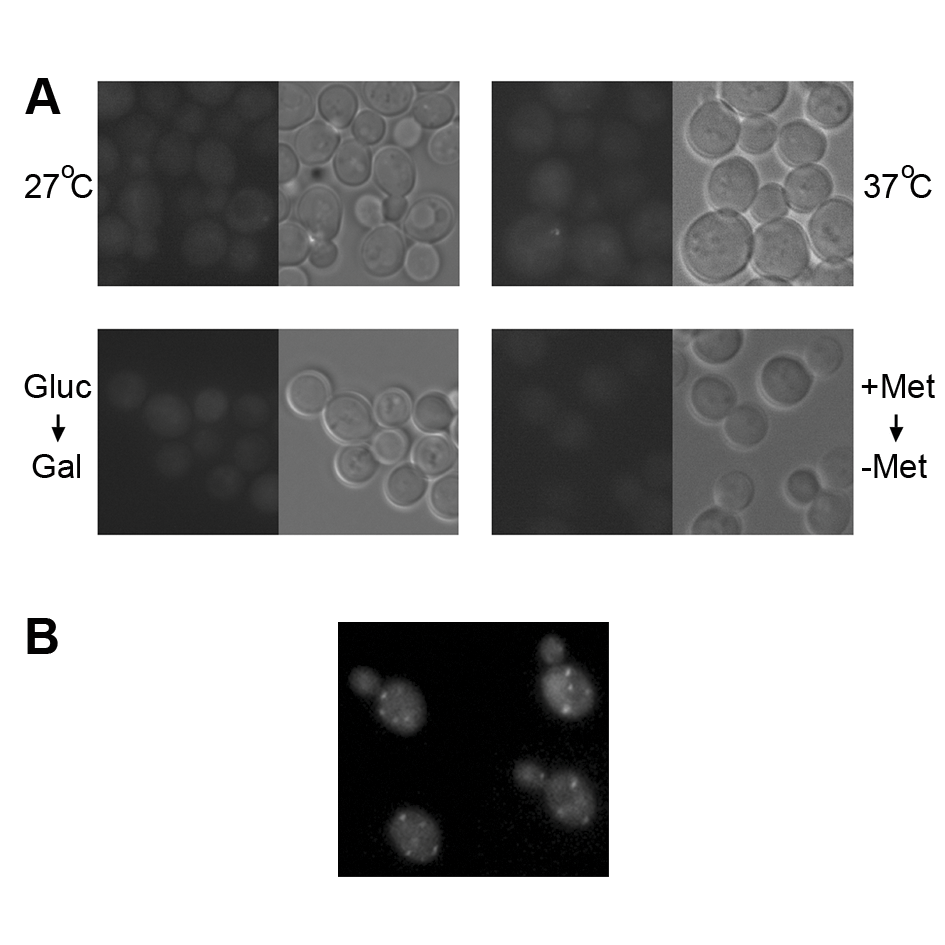

Supplement: Figure S1 — (A) Fluorescence microscopy of cells expressing Trs20-GFP (pUG-TRS20, see Table S1). No specific localization pattern could be observed under different conditions. (27°C, 37°C, a shift from glucose to galactose containing media or from media containing 1 mM methionine to media without methionine). Flourescence microscopy (left) and light microscopy (right) in each panel. (B) Fluorescence microscopy of cells expressing the TRAPP subunit Bet3 tagged with GFP (Δbet3 + pUG23-Bet3) shows the typical punctate Golgi pattern. (TIF) [file pone.0041408.s001.tif]

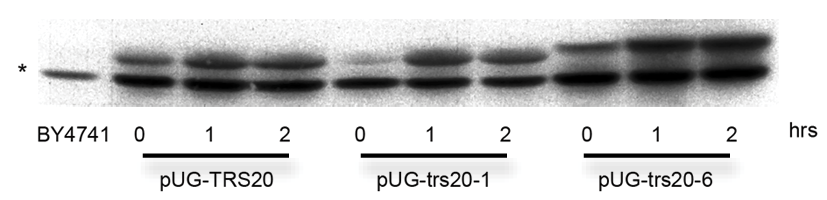

Supplement: Figure S2 — Western analysis of pUG-trs20-1 or pUG-trs20-6 mutants. Δtrs20 cells transformed with the plasmids pUG23-Trs20, pUG23-trs20-1 or pUG23-trs20-6 were taken after incubation at the permissive temperature (27°C, 0) or after 1 or 2 hrs at 37°C, total protein extracts were separated by SDS-PAGE, blotted and immunodetected with an anti-GFP antibody. A protein extract from non-transformed BY4741 cells was run in parallel. * marks a non-specific band. (TIF) [file pone.0041408.s002.tif]

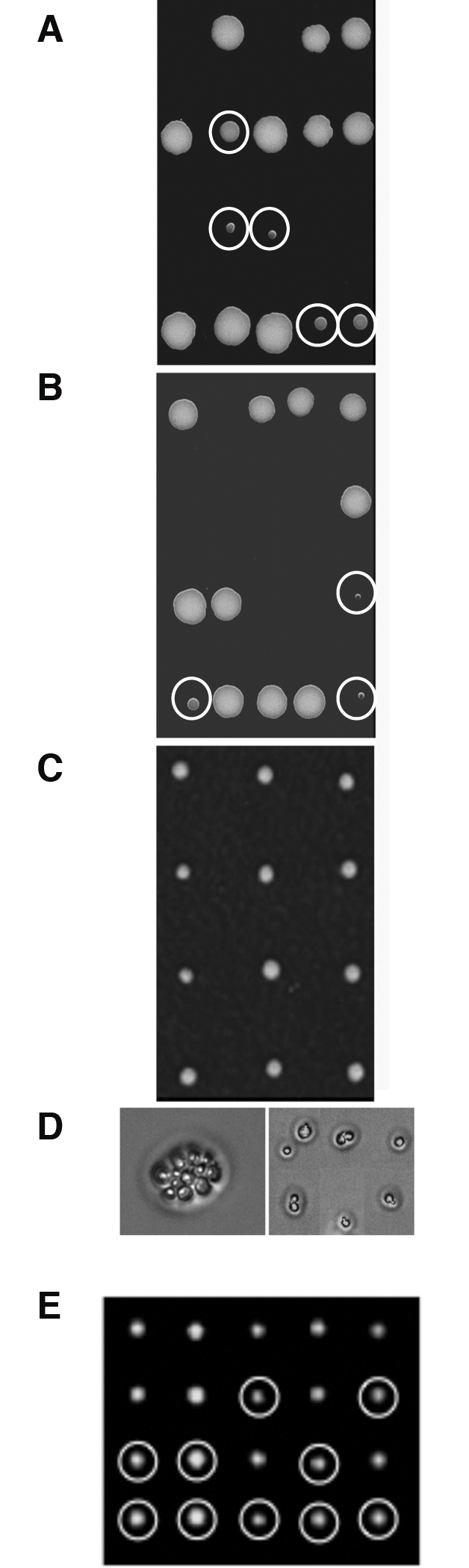

Supplement: Figure S3. — pUG-trs20-1 and pUG-trs20-6 spores show poor growth following germination. Heterozygous Trs20/Δtrs20 diploids carrying (A) the pUG23-trs20-1 or (B) the pUG23-trs20-6 plasmid were sporulated and subjected to tetrad analysis. The circled spore clones were Kan+, His+, all others were kan- (the Trs20 gene is deleted by replacement with a kanamycin resistance gene and the pUG23 plasmid carries a histidine selectable marker). The plates were incubated for 4 days at 27°C. (C) Tetrad analysis of Trs20/Δtrs20 diploids expressing a plasmid-borne wild type copy (pUG23-Trs20) of the Trs20 gene. The plates were incubated for 2 days at 27°C. (D) The defect is not due to a problem in germination. The spores that did not give rise to visible colonies were checked under the microscope and approximately 90% of them gave rise to microcolonies (left panel). Dissection of these microcolonies showed that the spores had undergone 3–5 replications before arresting growth (right panel). (E) Tetrad analysis of Trs85/Δtrs85 diploids. The circled spore clones were Kan+ (the Trs85 gene is deleted by replacement with a kanamycin resistance gene). (TIF) [file pone.0041408.s003.tif]

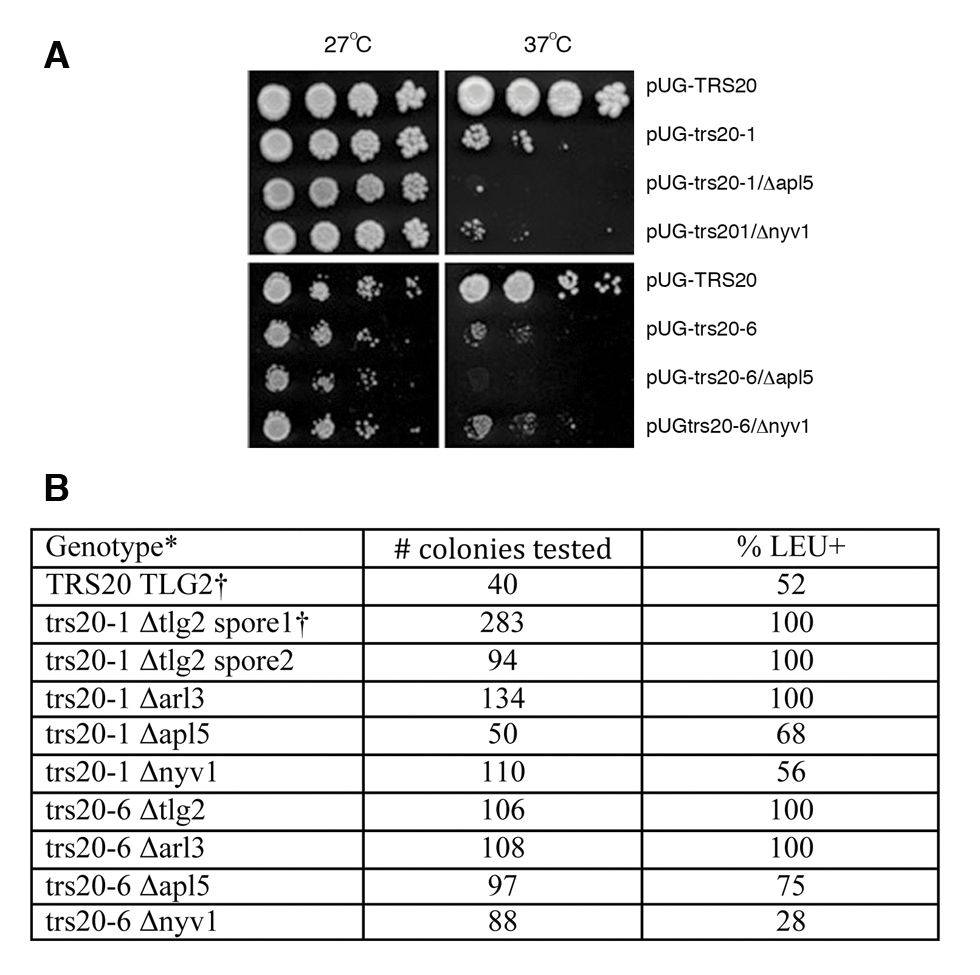

Supplement: Figure S4 — Genetic interactions of pUG-trs20-1 and pUG-trs20-6 . (A) Serial dilutions of pUG-TRS20, pUG-trs20-1, pUG-trs20-6 and the indicated double mutant cells replica plated on YPD were incubated at the indicated temperature for 3 days. No further growth of the Trs20 mutants combined with Δapl5 was observed even after longer incubation times at 37°C (data not shown). (B) Synthetic lethal interaction of pUG-trs20-1 and pUG-trs20-6 with Δtlg2 and Δarl3. Double mutants of pUG-trs20-1 or pUG-trs20-6 with the gene-of-interest were generated by mating and tetrad analysis (see Materials and Methods for details). All clones contained the mutated Trs20 gene on a plasmid with a HIS selectable marker and the WT gene on a plasmid with a LEU selectable marker. Following growth in medium containing leucine, individual colonies were tested for retention of the LEU-containing plasmid carrying the WT Trs20 gene, YCplac111-TRS20 (% LEU, see Materials and Methods for details). *The genotypes were verified by PCR. † Meiotic products from the same tetrad. The genotype of the TRS20-TLG2 cells was inferred from the kanamycin sensitive phenotype (deleted genes carry a Kan resistance gene). Three additional kanamycin-sensitive clones tested showed approximately 40% retention of the LEU selectable marker (data not shown). (TIF) [file pone.0041408.s004.tif]

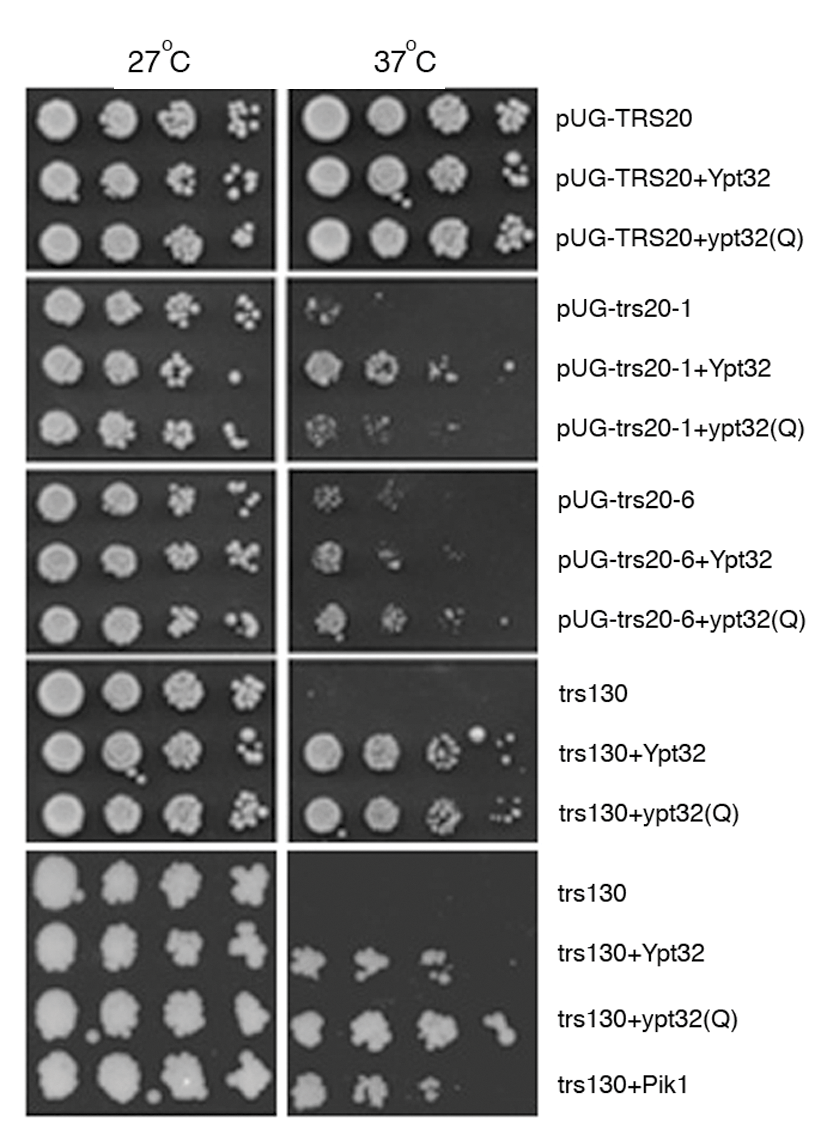

Supplement: Figure S5 — Suppression analysis of pUG-trs20-1 , pUG-trs20-6 and trs130-HAts cells. Cells transformed with low copy number plasmids carrying pUG36-Ypt32 or pUG36-ypt32Q72L (Ypt32(Q)) were serially diluted, replica plated on YPD and incubated at the indicated temperature for 3 days. Pik1p, expressed from a high copy number plasmid, suppresses the temperature-sensitive phenotype of trs130-HAts cells. (TIF) [file pone.0041408.s005.tif]

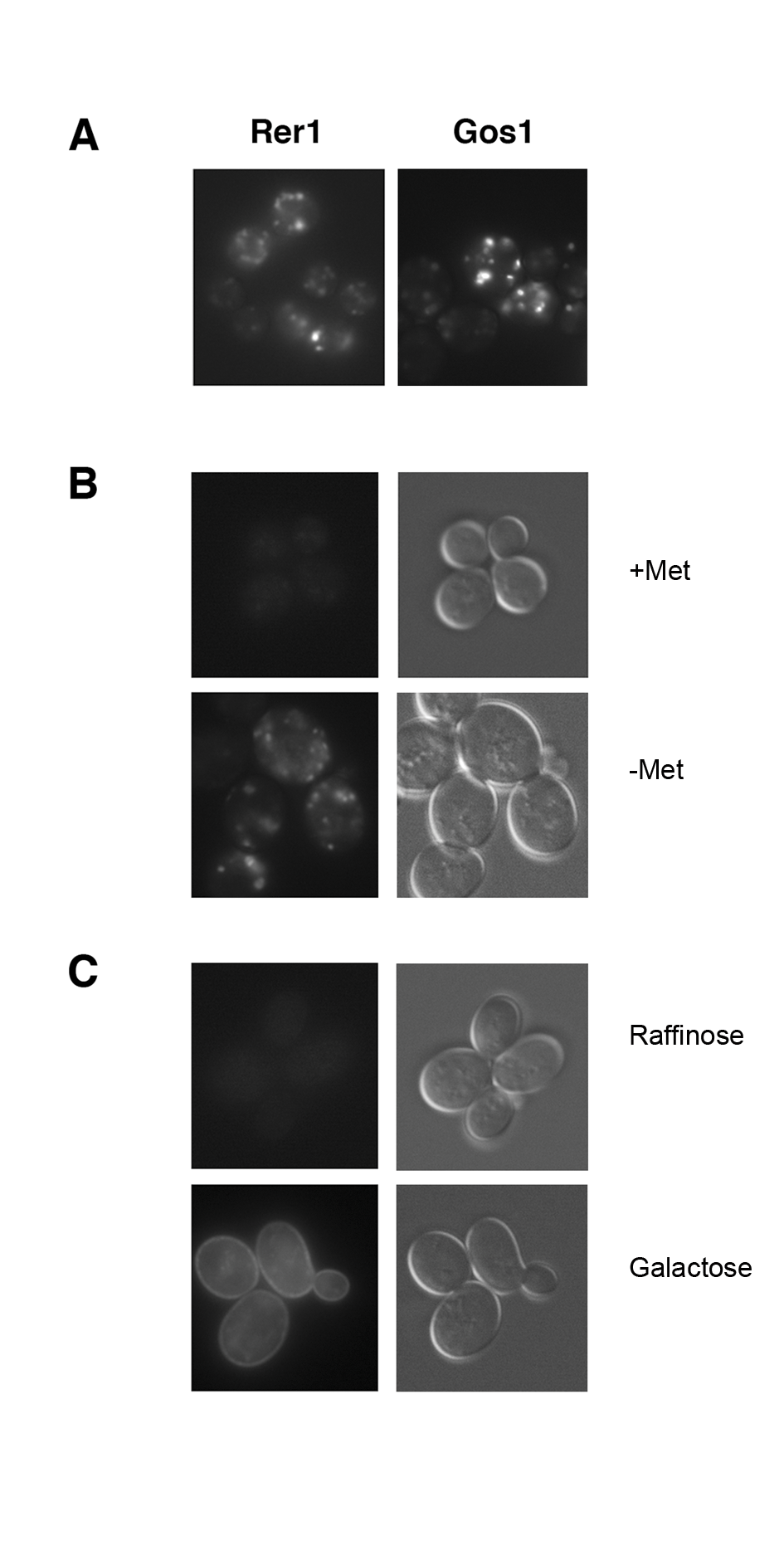

Supplement: Figure S6 — The YCplac-trs20-6 mutant does not affect Rer1p, Gos1p or Gap1p trafficking. (A) Rer1p-GFP and Gos1p-GFP under steady state conditions (27°C). (B) Induction of Gos1p-GFP expression by shifting cells from medium containing 1 mM methionine to medium without methionine. Left panels, fluorescent microscopy, right panels DIC images. (C) Induction of Gap1p-GFP expression by shifting cells from raffinose to galactose. Left panels, fluorescent microscopy, right panels DIC images. Experiments were performed as described in Materials and Methods, see main text and Figures 2, 3. (TIF) [file pone.0041408.s006.tif]

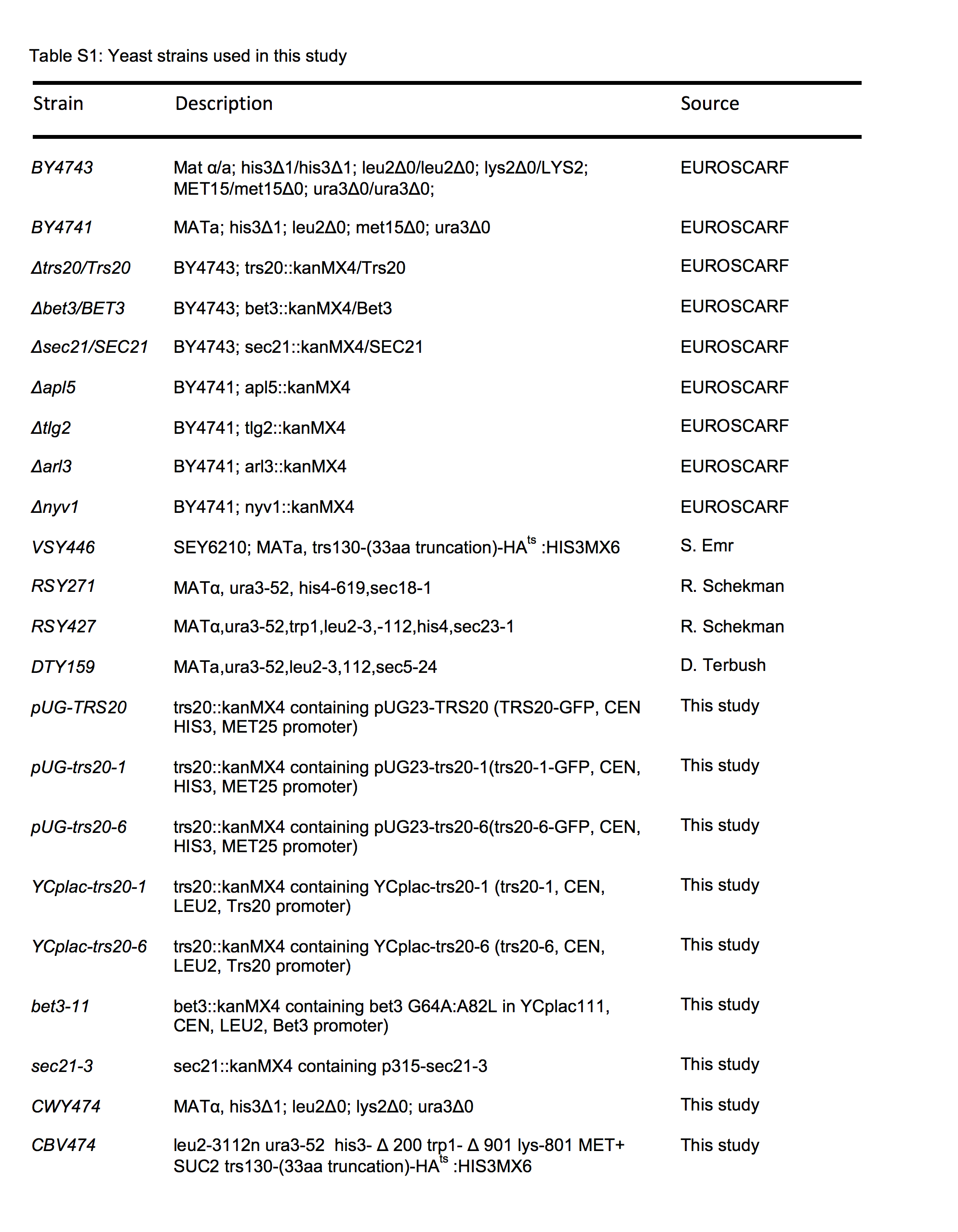

Supplement: Table S1 — Strains used in this study. (DOC) [file pone.0041408.s007.doc]

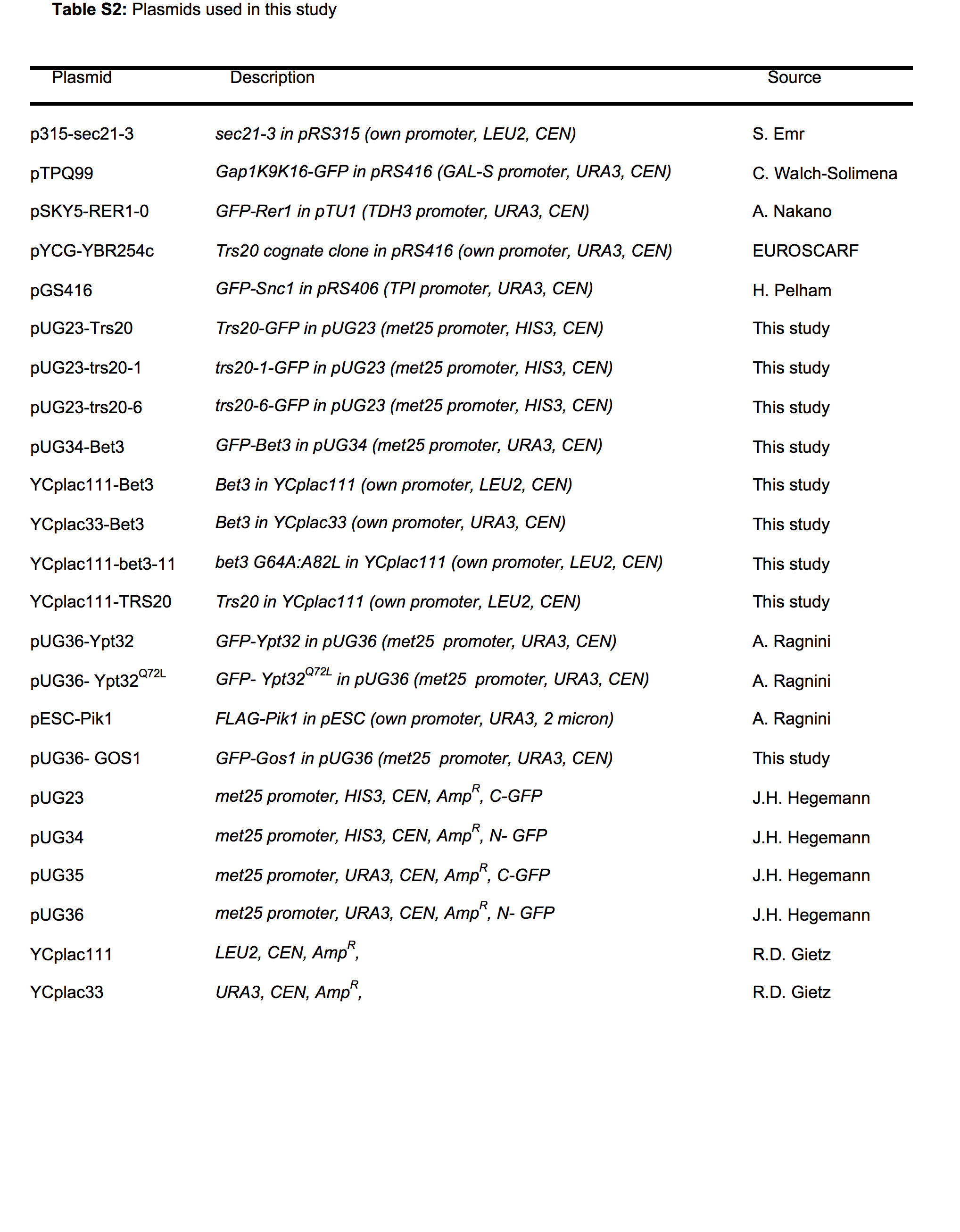

Supplement: Table S2 — Plasmids used in this study. (DOC) [file pone.0041408.s008.doc]
